# How I treat diffuse large B-cell lymphoma

**DOI:** 10.1016/j.esmoop.2022.100750

**Published:** 2023-01-10

**Authors:** T. Melchardt, A. Egle, R. Greil

**Affiliations:** 1 IIIrd Medical Department at the Paracelsus Medical University Salzburg, Cancer Center, Salzburg; 2 Salzburg Cancer Research Institute, Salzburg; 3 Austrian Group for Medical Tumor Therapy (AGMT), Salzburg; 4 Cancer Cluster, Salzburg, Austria

**Keywords:** diffuse large B-cell lymphoma, interim PET, polatuzumab vedotin, CAR-T cells

## Abstract

Diffuse large B-cell lymphoma (DLBCL) is usually treated with chemoimmunotherapy in curative intention at initial diagnosis. Novel agents have improved the prognosis of high-risk patients in the front-line and relapsed setting and more accurate prognostic tools enable less intensive treatment for low-risk patients, while maintaining their good prognosis. Here, we summarize our approach to DLBCL patients in the first-line setting according to their risk profile and other common challenges in clinical practice. We recommend an abbreviated course of chemoimmunotherapy in low-risk patients and a negative interim positron emission tomography. For patients with higher-risk disease, a new combination treatment with polatuzumab vedotin has been approved and is a new option in these patients. We also discuss our approach to patients with high risk for subsequent central nervous system involvement, with leg-type lymphoma or with severe comorbidities.

## Highlights

• Several novel drugs have improved first-line and second-line treatment of DLBCL in clinical practice.
• De-escalation of low-risk patients reduces acute and late side-effects.
• New treatment concepts such as CAR-T cells and bispecific antibodies will further improve the outcome of high-risk patients.

## Introduction

Diffuse large B-cell non-Hodgkin’s lymphoma is the most common aggressive B-cell lymphoma and accounts for ∼30% of all lymphomas. The median age at first diagnosis is about 70 years and between 50% and 60% of all patients are cured with rituximab-based chemoimmunotherapy in the first-line setting.^1^ Treatment causes substantial morbidity due to acute and long-term toxicity and a treatment-related mortality between 2% and 8%. Relapsed or refractory disease is typically observed within the first 2 years after diagnosis and requires more intensive treatment with high-dose chemotherapy, CD-19-directed chimeric antigen receptor (CAR)-T-cell or experimental therapy in eligible patients.

**Figure 1:**
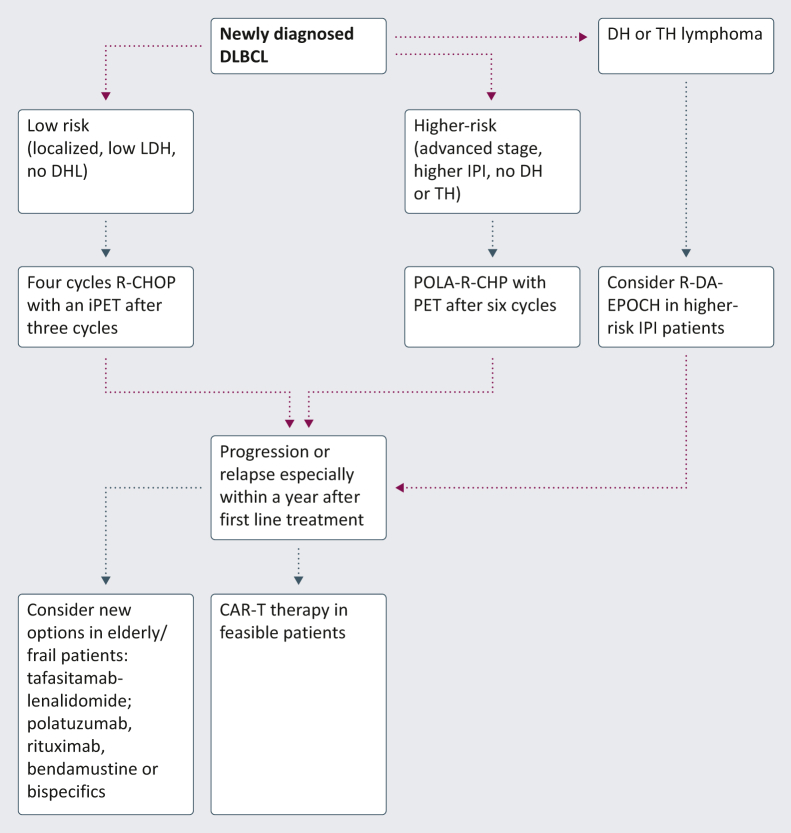
My approach to DLBCL outside a clinical trial. CAR-T, chimeric antigen receptor T cell; DHL, double-hit lymphoma; DLBCL, diffuse large B-cell lymphoma; iPET, interim positron emission tomography; IPI, international prognostic index; LDH, lactate dehydrogenase; POLA-R-CHP, polatuzumab, vedotin, rituximab, cyclophosphamide, doxorubicin and prednisone; R-CHOP, rituximab, cyclophosphamide, doxorubicin, vincristine and prednisone; R-DA-EPOCH, rituximab-dose-adjusted, etoposide, prednisone, vincristine, cyclophosphamide and doxorubicin; TH, triple-hit.

Multiple clinical trials and analyses have been carried out in the last decade and resulted in new options in this disease. Here, we present and discuss our clinical approach outside clinical trials (Figure 1).

## Diagnosis, staging and treatment planning

At initial work-up, we carry out a complete physical examination, blood count, renal and liver function, albumin, β_2_-microglobulin and quantitative immunoglobulins. A contrast-enhanced computer tomography (CT) scan of neck, thorax and abdomen is usually already available at first presentation. In patients with CT-confirmed advanced stage at initial diagnosis, we usually omit an additional positron emission tomography (PET) scan outside a clinical trial due to the lack of additional clinical consequence. We carry out a bone-marrow biopsy including a t(14;18) translocation analysis in all patients to exclude lymphoma infiltration, especially of an unknown low-grade component.

Nevertheless, although some new risk scores such as the National Comprehensive Cancer Network international prognostic index (NCCN-IPI) have been published in the last decade, we still use the IPI, which was first reported in 1993, for risk stratification at initial diagnosis. Despite this long time, all current algorithms of treatment de-escalation and escalation are based on this clinical risk assessment. Furthermore, we use the central nervous system (CNS)-IPI to estimate the individual prognosis overall and the risk of CNS involvement.^2^ Patients with a risk of CNS involvement of 10% or higher or neurological symptoms are further evaluated using cerebral magnetic resonance imaging and cytology and flow cytometry of the cerebrospinal fluid. Different sites of involvement and their numbers were discussed in the past as risk factors for CNS involvement, but in the largest dataset, which is the base of the CNS-IPI, only renal and adrenal gland involvement was independently associated with CNS involvement. In addition, while these risk factors are predictive, apart from the effect of rituximab in lowering the brain relapse rates, a recent meta-analysis of 36 studies including 5 randomized studies could not provide evidence for a benefit of intrathecal or intravenous therapy in the counteraction of these risk factors.^3^ However, NCCN and European Society for Medical Oncology (ESMO) guidelines still recommend such prophylaxis under certain conditions and thus these decisions have to be discussed with the patient ‘case-by-case’.^4^

For assessment of comorbidities, all patients are assessed by echocardiography, electrocardiogram and brain natriuretic peptide before we consider anthracycline-based chemotherapy. We also assess human immunodeficiency virus (HIV), hepatitis B and C serology in all patients before treatment. We use entecavir or tenofovir in all patients with positive hepatitis core antibodies (anti-HBc) indicating previous infection regardless of the results of the viral load by quantitative PCR. If quantitative PCR is positive, we monitor the viral load during chemoimmunotherapy and the first year of the follow-up. Furthermore, we offer psychological support to all patients and families at initial diagnosis.

### Histological assessment at first diagnosis

The cornerstone of first diagnosis is the histological assessment of infiltrated tissue and we do not rely on fine-needle aspiration for lymphoma subtyping. Histological assessment includes a standard immunohistochemistry panel including common B- and T-cell markers and an analysis of the cell of origin and an MYC, BCL2 and BCL6 FISH analysis according to the World Health Organization classification of lymphoma of 2016 incorporating the cell of origin and the double- or triple-hit status.^5^ In case of relevant and predominant pleural or peritoneal effusion, we also clarify the human herpesvirus-8 status.

### First-line treatment of low-risk patients

Patients with low-risk disease defined by an IPI below 2 treated with rituximab-based chemoimmunotherapy have a favorable prognosis. In patients with limited disease, several randomized clinical trials have shown the equivalence of four cycles compared to six cycles of R-CHOP (rituximab, cyclophosphamide, doxorubicin, vincristine and prednisone) chemotherapy.^6^, ^7^, ^8^ We recommend all patients with limited-stage disease and an age-adjusted IPI of 0 and also some selected patients with only slightly elevated lactate dehydrogenase (LDH) levels and limited-stage disease an abbreviated treatment and an interim PET after three cycles. If this PET scan shows a Deauville score below 4, which is the case in >80% of the patients, we stop first-line treatment after four cycles of R-CHOP.

In patients with a positive interim PET, we try to distinguish between the small proportion of patients with primary refractory disease despite a low IPI and the group of patients with a response based on CT morphology, but persisting PET uptake. In the latter group and depending on the number of involved sites, we evaluate consolidation radiotherapy or prolongation of R-CHOP to a total number of six cycles followed by a new PET scan. In the small group of patients with progressive disease based on an increase of lesion size in the CT scan and increased uptake in the PET after three cycles of R-CHOP, we carry out a repeated biopsy to confirm the initial diagnosis and evaluate the patient for the optimal second-line treatment.

In patients with low-risk advanced-stage disease and an IPI of 1, we carry out six cycles of CHOP and eight cycles of rituximab followed by a final PET scan.

### First-line treatment of higher-risk patients

The majority of patients do not fulfill the criteria for abbreviated therapy and have no low-risk disease. A recent randomized clinical trial showed the superiority in progression-free survival and reduced need for further treatment lines with the use of polatuzumab vedotin instead of vincristine incorporated in the standard R-CHOP regimen in patients with newly diagnosed diffuse large B-cell lymphoma (DLBCL) with an IPI >2.^9^ Therefore, polatuzumab vedotin was approved in 2022 for this use in Europe and is our preferred regimen in these patients.

To improve the tolerability of polychemotherapy, we use an neurokinin 1 receptor antagonist in addition to metoclopramide and serotonin receptor antagonists receptor antagonists to reduce nausea and we prescribe filgrastim in all patients. In very young patients, we often recommend shorter courses of filgrastim due to excessive bone pain and marked leucocytosis. In standard-risk patients, we do not use antibiotics as primary prophylaxis, but recommend valaciclovir to all patients with a herpes zoster reactivation in the last 6 months. In patients with cardiovascular risk factors and age >60 years, we use liposomal anthracyclines despite the fact that better safety profile was not proven in a randomized clinical trial in lymphoma patients.^10^

We carry out a clinical examination before every treatment cycle to assess the clinical response and do a CT scan after three cycles and a PET scan after completion of treatment. Despite the difficulties in the availability of PET scans, we carry out earlier PET scans in patients with signs of early progression including no improvement of lymphoma-related symptoms or initially increased LDH due to the possible benefit of alternative treatment strategies including early CAR-T-cell use.

Patients with a response based on CT morphology, but persisting isolated PET uptake, are referred for radiotherapy as consolidation or are evaluated for a biopsy of the residual lesion if feasible. After radiotherapy we repeat the PET scan after 3 months. Patients with large extranodal masses or bulky disease at first diagnosis are evaluated for consolidative radiotherapy also with PET-negative disease.

Patients with a double- or triple-hit lymphoma defined by an MYC and BCL2 and/or BCL6 rearrangement detected by FISH have a worse prognosis independent from their basic IPI risk. We usually treat these patients with a dose-adjusted EPOCH (etoposide, prednisone, vincristine, cyclophosphamide and doxorubicin) regimen based on a phase II trial in patients with an MYC rearrangement and a meta-analysis based on 11 retrospective studies showing favorable results with this more intensive treatment regimen.^11^^,^^12^ Nevertheless, we acknowledge that some recent retrospective analyses have not shown a benefit of this more intensive regimen in double-hit lymphoma patients and therefore, this difficult issue should be discussed on a case-by-case basis with the patient.

In clinical practice, we also face other high-risk situations defined by frail or highly symptomatic patients or special situations such as leg-type lymphoma, HIV-associated DLBCL or circumstances, which are often associated with CNS involvement. Our standard of care for these patients is summarized in Table 1.

**Table 1:** Special situations

High CNS-IPI
MRI and liquor assessment using cytology and flow cytometry in patients with CNS-IPI >10% to exclude CNS involvement is recommended. Consider CNS-directed treatment followed by consolidative autologous stem cell transplant in patients with cerebral involvement.
Leg-type lymphoma
Consolidative radiotherapy after systemic treatment is recommended, avoid extensive surgery.
Frail patients
Off-label use of bendamustine, rituximab and polatuzumab vedotin and tafasitamab with lenalidomide should be considered in patients not eligible for anthracycline-based chemotherapy.
Patients with high-volume and symptomatic disease
In patients highly symptomatic due to high-volume disease, we often consider immediate start of polychemotherapy instead of a pre-phase treatment with corticosteroids. Strict prophylaxis of tumor lysis syndrome and close monitoring of the serum parameters are mandatory in these patients.
Patients with HIV-associated DLBCL
It is important to start (or continue) an effective ART, watching drug–drug interactions. In patients with CD4 <50/μl, the use of rituximab is individualized to lymphoma and infection risks. In patients with CD4 count >50/μl, our former standard regimens R-CHOP or R-EPOCH are now challenged due to the better efficacy of polatuzumab vedotin.

ART, antiretroviral therapy; CNS, central nervous system; DLBCL, diffuse large B-cell lymphoma; HIV, human immunodeficiency virus; IPI, international prognostic index; MRI, magnetic resonance imaging; R-CHOP, rituximab, cyclophosphamide, doxorubicin, vincristine and prednisone; R-EPOCH, rituximab, etoposide, prednisone, vincristine, cyclophosphamide and doxorubicin.

### Survivorship and follow-up after first-line treatment

If patients achieve a PET-negative remission after initial treatment, further follow-up is planned. Unfortunately, despite cessation of cytotoxic treatment and complete remission, many patients suffer from fatigue, polyneuropathy or anxiety. Therefore, we refer patients with anxiety as early as possible for psychological support and initiate psychotropic drugs if necessary. In patients with polyneuropathy, we recommend dose reductions of vincristine and symptomatic treatment with gabapentin or duloxetine despite the low level of evidence for a significant benefit. Furthermore, patients are referred for a stay in cancer rehabilitation clinics to improve these symptoms and quality of life.

During further follow-up, we avoid regular CT scans in asymptomatic patients due to the proven lack of efficacy according to the recommendations of the American Society of Hematology. We recommend a blood count, renal and liver function, LDH and a clinical examination every 3 months in the first 2 years after treatment and use chest X-ray and ultrasonography in patients with predominant mediastinal or retroperitoneal disease at first diagnosis. NCCN guidelines still include surveillance CT scans during the follow-up in patients with aggressive lymphoma, but we use this only in selected patients with a high risk of relapse.

### Relapsed or refractory disease

In patients with persisting PET-positive disease or new lymphadenopathy after first-line treatment, we carry out a new biopsy to confirm malignancy and the former diagnosis. Recently, two randomized clinical trials have shown the superiority of CAR-T-cell products over autologous stem cell transplant^13^^,^^14^ in patients with relapsed or refractory disease within 1 year after first-line treatment, thus substantially changing the treatment preferences in this situation. An expansion of the existing European Medicines Agency (EMA) approval of these agents is expected in late 2022. Furthermore, new therapeutic antibodies such as polatuzumab vedotin in combination with bendamustine and rituximab and tafasitamab with lenalidomide are already approved in this setting and are especially used in patients not eligible for autologous stem cell transplant or CAR-T-cell therapy. Bispecific antibodies such as epcoritamab and glofitamab have also shown impressive overall response rates in heavily pretreated patients. For patients not recruitable to trials with these and other novel drugs, epcoritamab and glofitamab are already available and we use them in early access programs outside clinical trials.

To summarize, based on the data of the randomized trials, we recommend CAR-T cells in the second-line setting in patients feasible for this intensive treatment. For patients not eligible due to comorbidities, we prefer polatuzumab vedotin in combination with bendamustine, as long as polatuzumab vedotin has not been part of the first-line treatment, and rituximab or tafasitamab with lenalidomide over palliative chemotherapy.

## Outlook

Due to the availability of new agents and their advancement to first-line treatment as well as the ongoing research on the molecular background of DLBCL, we expect further improvement in the treatment in everyday practice. Circulating tumor DNA may complement the prognostic role of interim PET to identify low- and high-risk patients during the course of treatment and help us to guide treatment. Several new agents have now improved later treatment lines and the best sequence or combination treatment for different molecular subtypes may be identified in ongoing clinical and translational research.
